# The effectiveness of individual schema therapy in older adults with borderline personality disorder: Protocol of a multiple-baseline study

**DOI:** 10.1016/j.conctc.2019.100330

**Published:** 2019-01-24

**Authors:** D.A. Khasho, S.P.J. van Alphen, S.M.J. Heijnen-Kohl, M.A. Ouwens, A. Arntz, A.C. Videler

**Affiliations:** a GGz Breburg, Postbus 770, 5000 AT, Tilburg, the Netherlands; b Mondriaan, Postbus 4436, 6401 CX, Heerlen, the Netherlands; c University of Amsterdam, Postbus 15933, 1001 NK, Amsterdam, the Netherlands; d Tranzo Department, Tilburg University, Postbus 901535000 LE Tilburg, the Netherlands; e Tilburg University, Postbus 901535000 LE Tilburg, the Netherlands; f Vrije Universiteit Brussel, Pleinlaan 2 B-1050 Brussel, Belgium

**Keywords:** Multiple baseline case series, Schema therapy, Personality disorder, Borderline personality disorder, Older adults

## Abstract

**Background:**

The treatment of borderline personality disorder (BPD) has been examined extensively in adults up to the age of fifty in the past quarter of a century, but there is still a world to discover in treating BPD in older adults. The aim of the study is to investigate the effectiveness of schema therapy in older adults with BPD.

**Methods/design:**

A multiple baseline design is used in which participants are randomly assigned to baseline length. The primary outcome measure is assessed weekly and consists of the credibility of negative core beliefs. Secondary outcome measures are quality of life, psychological distress, early maladaptive schemas, schema modes, severity of BPD symptoms and meeting the criteria for BPD. Ten older adults (age > 60 years) with BPD are treated with schema therapy, with weekly sessions during one year. This treatment phase is preceded by a baseline phase varying from 4 to 8 weeks. After treatment, there is a 6-month follow-up phase with monthly booster sessions.

**Discussion:**

To our knowledge, this is the first empirical study of the effectiveness of psychotherapeutic treatment for BPD in older adults. Because of the different manifestation of BPD in later life, besides section II DSM-5 criteria, the alternative, dimensional model for personality disorders of DSM-5 is used to assess BPD in older adults.

**Trial registration:**

The Netherlands National Trial Register NTR7107. Registered 11 March 2018.

## 1 Introduction

Borderline personality disorder (BPD) is a lifelong pattern of instability in interpersonal relationships, self-image, and affect [1]. It also features impulsivity and self-harming behavior. The prevalence of BPD is estimated to be around 1–2% [2]. In older age groups (>60 years) the prevalence of BPD may decrease [3]. Although the prevalence can be lower, there is still a substantial group of older adults that meet the criteria for BPD, and core features of BPD, especially emotional dysregulation and disturbed interpersonal relationships persist into old age [4]. These BPD features are associated with psychosocial impairment in older adults [5]. BPD in later life differs in presentation from that seen in younger adults [6]. Lower levels of impulsivity and identity disturbance are observed, in contrast with increased feelings of emptiness, and somatic symptoms and complaints. Also, the self-harming behavior may change, e.g., non-compliance with a medical treatment instead of auto-mutilation. As a consequence of the changes in the manifestation of BPD in later life, it has proven difficult to diagnose personality disorders adequately in older adults [7,8]. In large-scale item response theory analyses, less personality disorder criteria were identified in older people as compared to younger people [9] and even 29% of personality disorder criteria showed unacceptable high measurement errors in older people [9]. Thus, the description of BPD-traits in the Diagnostic and Statistical Manual of Mental Disorders [2] might lead to an underestimation of the ‘true’ prevalence of BPD at older age. This calls for a broader view on the behavioral expression of borderline pathology in older adults.

An alternative approach for personality disorders is described in section III of the DSM-5 [2]. In this alternative model, the diagnosis of personality disorders is characterized by impairments in personality functioning (Criterion A) and pathological personality traits (Criterion B), which are dimensionally assessed. Dimensional models like this alternative DSM-5 model seem to be more fruitful than the categorical ones, like section II of DSM-5, for assessing personality pathology in later life [8,10]. Increased scores on the pathological traits negative affectivity, disinhibition, and psychoticism are aspects of this alternative DSM-5 model that are correlated with the criteria of the DSM-5 section II model for BPD [11]. The construct validity of both the DSM-5 section III maladaptive trait domains [12] and the Severity Indices of Personality Functioning–Short Form (SIPP-SF) have been demonstrated in older adults as a measure to assess personality functioning [13] Therefore, in the current study we incorporate a broad diagnostic view in assessing BPD in older adults, employing both the current, categorical and the alternative, dimensional approaches, preventing missed diagnoses and refining the diagnostic process.

Research shows that there are different ways to adequately treat BPD, among which are dialectical behavior therapy, systems training for emotional predictability and problem-solving, mentalization-based treatment, transference-focused psychotherapy, and schema therapy [14]. Schema therapy (ST) is a form a treatment that seems to connect to the psychotherapy expectations of older adults, because it incorporates psychoeducation and because of its structured, skill-enhancing and problem-focused features [15]. ST is an integrative treatment, which draws on the cognitive-behavioral, attachment, psychodynamic, and emotion-focused traditions and can be adjusted for treating BPD [16]. In this treatment model, early maladaptive schemas (EMS) are considered core elements of personality disorders. EMS are self-defeating emotional and cognitive patterns established in childhood and repeated throughout life [17]. When a maladaptive schema is activated, associated painful emotions arise; early in life, patients have developed coping strategies to deal with these emotions. A schema mode is a combination of activated schemas and coping strategies, which describes the current emotional, cognitive, and behavioral state a patient is in. Modes are states which can change quickly, while schemas are more like traits, rigid and enduring. Cognitive interventions, experiential techniques, and the therapeutic relationship are important elements in schema therapy. The goal of treatment is to decrease the impact of the EMS and to replace maladaptive schema modes with more healthy alternatives so that patients succeed in getting their core emotional needs met [18].

ST has been proven effective in treating younger and middle aged adults with personality disorders [19,20]. There is emerging proof that ST can also be effective for older adults with personality disorders [15,21]. A recent study showed that ST is an effective treatment for older adults with cluster C personality disorders [21]. This study was the first test of the effectiveness of any psychotherapy for personality disorders in older adults, using a multiple baseline design. Results revealed significant linear trends during the treatment phase, but not during baseline and follow-up. Seven of eight patients remitted from their personality disorder diagnosis.

The aim of the current study is to investigate whether ST can be an effective treatment for older adults with BPD. Successful treatment of BPD in older adults can improve well-being later in life [22] and can reduce the complications of treatment of other psychiatric disorders [23].

## 2 Present study

The aim of this study is to investigate the effectiveness of individual ST as a treatment for older adults with BPD. The research question is whether older patients with BPD benefit from ST. The primary objective is to study the effect of ST on the strength of negative core beliefs in older adults with BPD. We hypothesize that ST diminishes the strength of these negative core beliefs.

Secondary objectives are improvement of quality of life, reducing psychological distress, decreasing EMS and maladaptive schema modes, and reducing the severity of BPD symptoms. In particular, we hypothesize that participants will no longer meet the criteria for BPD, employing both the categorical and the alternative model for personality disorders of DSM-5.

## 3 Methods

### 3.1 Study design and procedure

This study is a multiple baseline case series design [24]. We chose this design for several reasons. A multiple-baseline case series design offers experimental control over time versus intervention effects, which is an important advantage over an open trial. Like an RCT, a multiple-baseline design can demonstrate significant change and that this change is the result of the intervention and not of time [25]. Secondly, an advantage of this design over a randomized controlled trial (RCT) is that this design requires fewer participants; participants act as their own controls, thus increasing power [26].

A multiple-baseline design requires a dependent variable that is frequently assessed and highly sensitive to change, in order to assess the time and intervention effects [25]. Such a variable should represent a core aspect of the disorder that is addressed by the treatment. The frequent assessments make it possible to distinguish time from treatment effects and allow that each case is its own control. In other words, the frequent assessments of the central variable compensate for the small number of participants. As central variable, we chose the strength of belief participants have in their personal core beliefs, which they view as central to their PD problems. This idiosyncratic measure represents the EMS that are assumed to underlie the patient's personality disorder problems according to the schema therapy model [16].

The design consists of three phases. First, a baseline phase varying in length from four to eight weeks. No therapeutic interventions will be applied during this phase. The second phase is the treatment phase and consists of ST for BPD according to the model of Young and colleagues [17]. Precise methods for the treatment of BPD using ST is described in Arntz & van Genderen [27]. The third and final phase is a six months follow-up with monthly booster sessions.

The length of the baseline phase is randomized across participants to increase the internal validity. Randomization of participants over variations in baseline length increases the experimental control over differentiating time from treatment effects. Baseline varies between four to eight weeks and will be randomly assigned by an independent colleague using a lottery system. The lottery system consists of five different possible outcomes (4, 5, 6, 7, and 8 weeks). Each different outcome is represented two times, making a total of ten outcomes. There is no replacement after each draw, so different baseline lengths are evenly distributed among participants.

The duration of the treatment phase is 52 weeks and is estimated to be sufficient to accomplish meaningful results [21]. During the treatment phase, the therapist decides when to introduce the different techniques, according to the ST model [16,27] and participants’ case conceptualization.

The follow-up phase lasts six months and includes monthly booster sessions in which the aim is to maintain the acquired ST knowledge and skills.

### 3.2 Participants and screening procedure

The participants are ten patients from the department of geriatric psychiatry of Breburg and from the department of geriatric psychiatry of Mondriaan, both mental health institutes in the Netherlands.

Four inclusion criteria must be met: 1) a primary diagnosis of BPD, assessed with the Structured Clinical Interview for DSM-5 Personality disorders (SCID-5-P; [28]). In this semi-structured interview experiences in early adulthood will be emphasized in order to increase the validity of the diagnosis. Participants who meet the subthreshold level of the diagnostic criteria of BPD are also included, given the different behavioral expression of BPD in older adults (as argued in the introduction), but only if there are increased scores on the traits negative affectivity, disinhibition, and psychoticism of the Personality Inventory for DSM-5 (PID-5; [29]). These three traits of the alternative model of DSM-5 are correlated with the DSM-5 section II diagnosis of BPD [30]. A mean score of 2 or higher is seen as increased [31]. The PID-5 will be administered to both the participant and a close relative; 2) absence of chronic somatic comorbidity which seriously affects daily life functioning, demonstrated by a minimum Barthel-score of 15 [32]; 3) minimum age of 60 years; 4) willingness to participate in the study.

Exclusion criteria are severe depression, bipolar disorder, psychotic disorder (other than transient stress-related psychosis, if this overlaps with criterion 9 of BPD), IQ under 80, substance dependence that needs clinical detoxification (participation is possible after successful detoxification), and neurocognitive disorder expressed by a MMSE-score below 25 [33]. All these factors are taken into account during the multidisciplinary clinical assessment at the particular mental health institute. These factors are labeled exclusion criteria in consideration of their possible disturbing effect on treatment. No other treatment is allowed and preferably the use of psychiatric medication is kept constant during the study and is at least monitored using single session registration forms.

Patients who meet these criteria are approached by the first author to participate in the study.

### 3.3 Instruments and outcome measures

#### 3.3.1 Primary outcome measures

The primary outcome measure is the weekly assessed credibility of idiosyncratic beliefs. These beliefs are evoked by a semi-structured procedure during the baseline phase. Each participant formulates three to five idiosyncratic, dysfunctional beliefs they consider to be central to their personality disorder. Participants will rate the degree they believe each statement on a visual analog scale (VAS) from 0 to 100% credibility. The core beliefs are rated weekly during baseline, treatment and follow-up phases, at the start of each session. The therapist will not be present when participants fill in the VAS to assure integrity. Filled-in forms will be given to the therapist in a closed envelope and passed on to the research team.

#### 3.3.2 Secondary outcome measures

*Quality of life* is assessed by the Dutch brief version of the World Health Organization Quality of Life (WHOQOL-BREF; [34]). The WHOQOL-BREF is a self-report measure that consists of 26 items, which are rated on a 5-point Likert scale. Research in two samples of older adults, with mean ages of 73 and 76 years, showed good reliability and satisfactory construct validity [35].

*Psychological distress* is measured by the Brief Symptom Inventory* (BSI; [36]). The BSI is a 53-item self-report measure, which is rated on a 5-point rating scale. The reliability and validity of the BSI are good, with reliability coefficients ranging from 0.68 to 0.91 [37], and the Dutch version of the BSI has been validated in older adults [38].

*Early maladaptive schemas* are measured with the Dutch Young Schema Questionnaire* (YSQ; [39]). The questionnaire consists of 16 subscales and includes 205 items, which are phrased as negative core beliefs and rated along a 6-point Likert scale. In a clinical sample with a mean age of 33.9 years (range 18–74) the YSQ showed good reliability and convergent and discriminant validity [40]. The YSQ measures EMS scales equally across age [41].

*Schema modes* are assessed by the Dutch Short Schema Mode Inventory* [42]. It consists of 14 subscales and includes 118 items which must be rated on a 6-point Likert scale. This questionnaire has acceptable internal consistencies (Cronbach α′s from 0.79 to 0.96) and moderate construct validity [42].

*Current severity of BPD* is assessed with the Dutch version of the BPDSI [43], a semi-structured interview which assesses the frequency and severity of BPD symptoms during the last three months. The BPDSI consists of nine sections, one for each of the DSM-criteria for BPD and appears reliable and internally consistent [43].

*Personality Disorder Diagnoses* are assessed with the SCID-5-P* [28], a semi-structured interview for DSM-5 personality disorder diagnoses. Personality disorder-criteria are rated on a 3-point scale as absent, sub-threshold or threshold. Reliability and validity are not yet available, but are expected to be similar to the Dutch version of the Structured Clinical Interview for DSM-IV Axis II disorders [44] as both instruments are almost similar. Inter-rater agreement appeared excellent in adults with an average age of 35.5 years (range 18–61), with a mean value of Cohen's kappa of .84 [45].

*Personality functioning* is measured with the Severity Indices of Personality Problems short form* (SIPP-SF; [46]), a self-report questionnaire aiming to measure the severity of the generic and changeable components of personality disorders. The SIPPF-SF consists of 60-items and five domains: self-control, identity integration, responsibility, relational capacities, and social concordance. Participants are asked to answer on a 4-point scale to what extent they agree with the statement presented, referring to the past three months. A study concerning the construct validity of the SIPP-SF for older adults demonstrated a structure of five higher order domains of personality functioning [13].

*Dimensional assessment of pathological personality traits:* The Personality Inventory for DSM-5* is a 220-item self-report questionnaire developed to assess personality traits in line with the dimensional perspective of personality pathology as advocated in Section-III of DSM-5 [47]. The original five-factor structure of the PID-5 has been confirmed in the Dutch version, and it has adequate reliability and convergent and discriminant validity [29].

These secondary outcomes will be measured four times: at the start of treatment and after six, twelve, and eighteen months, except for the SCID-5-P which will be administered twice (before and directly after the treatment phase).

### 3.4 Ethical issues and treatment integrity

The study procedure was reviewed and approved by the internal ethical committees of Breburg and Mondriaan, and METC Brabant, an independent ethical committee. All participants will be informed by the principal investigator, both orally and by letter, and sign an informed consent before the start of the study. Most of the measures used for inclusion and study outcomes are a standard part of routine outcome monitoring and standard diagnostic procedures at Breburg and Mondriaan (these measures are marked with an asterisk [*]). Two diagnostic instruments are added to this standard battery: the World-Health-Organization-Quality-Of-Life, brief version (WHOQOL-BREF, which measures quality of life) and the Borderline Personality Disorder Severity Index (BPDSI, which assesses the frequency and severity of BPD symptoms). ST can be considered a safe treatment as it has been found effective for BPD in younger adults, and it is increasingly used as a treatment in older adults in clinical practice; moreover, the effectiveness of ST has been established in older adults with cluster C personality disorders [21].

Treatment integrity is secured in five ways. All therapists in this study are well trained and educated in ST and are (candidate) members of the Dutch schema therapy association. Second, therapists can consult each other. Third, all therapists receive a training in which the study design and ways of reporting the sessions are made explicit. Furthermore, each session is registered using a tailor-made session registration form to describe the applied ST intervention. And finally, a random sample of five recorded sessions per therapy is rated for adherence to ST, using the Schema Therapy Rating Scale [48].

## 4 Statistical and power analysis

A multiple baseline design is, just like an RCT, capable of demonstrating the occurrence of change over time as a result of an intervention [49]. The fact that participants serve as their own controls and that the primary outcome is measured frequently can compensate for the potential loss of power. As an indication, it is possible to detect a large effect size (d > 1.5; power > .80) with four participants with twenty measurements each [24]. Due to the design of the current study, it is to be expected that the number of participants and measurements necessary to obtain such power will be exceeded.

Statistical analysis will be performed using a time series analysis based upon mixed regression. This type of analysis is possible because of the sufficient length of the treatment phase [25,26]. Mixed regression analysis will be used to explore the differences between treatment and the follow-up phase compared to the baseline phase.

## 5 Discussion

To our knowledge, this is the first empirical study of the effectiveness of psychotherapeutic treatment for BPD in older adults. In recent years there has been a shift in thinking about treatment possibilities in older adults with personality disorders [50]. Therapeutic nihilism has been replaced by a more optimistic and promising view that psychotherapy can make a difference for older adults who suffer from personality disorders and that it is possible to enhance their quality of life and sense of meaning in life. The treatment of BPD in younger adults has been examined extensively in the past quarter of a century, but there is still a world to discover in treating BPD in older adults. This study is a first step in the road ahead.

As stated, it is not straightforward to diagnose BPD in older adults. It is a challenging procedure because of the different manifestation of BPD in later life, and the fact that the DSM-description of BPD-traits might lead to an underestimation of the ‘true’ prevalence of BPD at older age. We have also employed a more dimensional model of BPD, in which, besides section II DSM-5 criteria, the pathological personality traits of the alternative model for personality disorders in section III of DSM-5 are used to diagnose BPD in older adults, for cases that are subthreshold BPD according to the criteria of section II of DSM-5. This diagnostic approach is not yet common in studies into BPD in younger age groups, but we think this is helpful to achieve comprehensive and meaningful results in studying BPD in later life.

A possible limitation of the present study is the length of treatment, which is set at 52 weekly sessions, followed by six monthly booster sessions. There is a great variation in the treatment dosage among different studies concerning ST and personality disorders, with the number of treatment sessions ranging from 78 up to 300 sessions [19,20,51]. The length of treatment in the present study is based on the fact that more recent studies show a reduction in length of treatment compared to earlier studies [19,52], and 52 sessions appeared effective in older adults with cluster C personality disorders [21].

A possible criticism could be the small sample size (*N* = 10), in particular compared to RCT studies. Yet the construction of a multiple baseline design makes it possible to acquire sufficient power to demonstrate treatment effects, even with a small *N*.

The results of this study will contribute to our understanding of treating BPD in older adults with ST and intends to provide beneficial information for the development of therapeutic interventions for older adults with BPD.

## Trial status

The trial started in August 2018 and data collection is expected to continue until September 2020.

## Competing interests

The authors declare that they don't have competing interests.

This research did not receive any specific grant from funding agencies in the public, commercial, or not-for-profit sectors.
